# Supplementary material for: Quantifying the direct and indirect protection provided by insecticide treated bed nets against malaria
Source: Nat Commun. 2023 Feb 8;14:676. doi: 10.1038/s41467-023-36356-9 (PMC9905482; doi:10.1038/s41467-023-36356-9)
Supplement: Supplementary file 1 — Supplementary Information [file 41467_2023_36356_MOESM1_ESM.pdf]

Supplementary Material: Modelling the impact of pyrethroid resistance on the mass community effect of insecticide treated nets

H. Juliette T. Unwin[1], Ellie Sherrard-Smith[1], Thomas S. Churcher[1], Azra C. Ghani[1]
[1]MRC Centre for Global Infectious Disease Analysis, Department of Infectious Disease Epidemiology, Faculty of Medicine, Imperial College London

Supplementary Methods: Imperial deterministic malaria model

We use a previously published malaria transmission model that fully incorporates the dynamics of *Plasmodium falciparum* transmission between human and vector hosts. The model is deterministic and similar to that presented in [1] and in [2].

The human model

Our model groups people within a population into compartments based on their age, which are denoted by the subscript i. At each point in time the age compartments can be in one of six infectious states – susceptible (S_i), treated clinical disease (T_i), untreated clinical disease (D_i), asymptomatic patent infection (A_i), sub-patent infection (U_i) and protected by a period of prophylaxis from prior treatment (P_i). People, or proportions of the population in each compartment, move between these states as shown in Figure A with the rates marked on the arrows and described below.

People are born into the first age compartment of this model as susceptible to infection, with new-borns possessing a level of maternally-inherited immunity that decays over the first six months of their lives. They move through the age compartments as expected due to natural aging. A proportion of each susceptible compartment is exposed to infectious bites from the mosquito vector as time passes. The hazard of infection for each compartment is determined by the force of infection (Λ_i), which is a function of the compartmental pre-erythrocytic immunity and biting rate, and the mosquito population size and level of infectivity.

Proportions of the infected compartment develop clinical disease or asymptomatic infection following a latent period (τ_E) (and move to compartments D or A) depending on the probability of acquiring clinical disease (ϕ_i), which is dependent on the compartments level of clinical immunity and is defined in equation (9). The proportion who develop clinical disease can be successfully treated (with a fixed probability f_T) and move to infection state *T*, or will not seek treatment (with probability $1 - f_T$) and move to infection state *D*. Proportions of the treated compartment then recover from infection at rate r_T and return to the susceptible infection state *S*. However, they retain a degree of drug-dependent partial protection from reinfection (modelled as a Weibull survivorship curve) which wanes over time [3].

The proportion of the untreated clinical disease state (D) compartment not receiving treatment recover to the asymptomatic infection state (*A*) at rate r_D. Death is not explicitly modelled. As parasite density is controlled, proportions of the asymptomatic state compartment progress to the

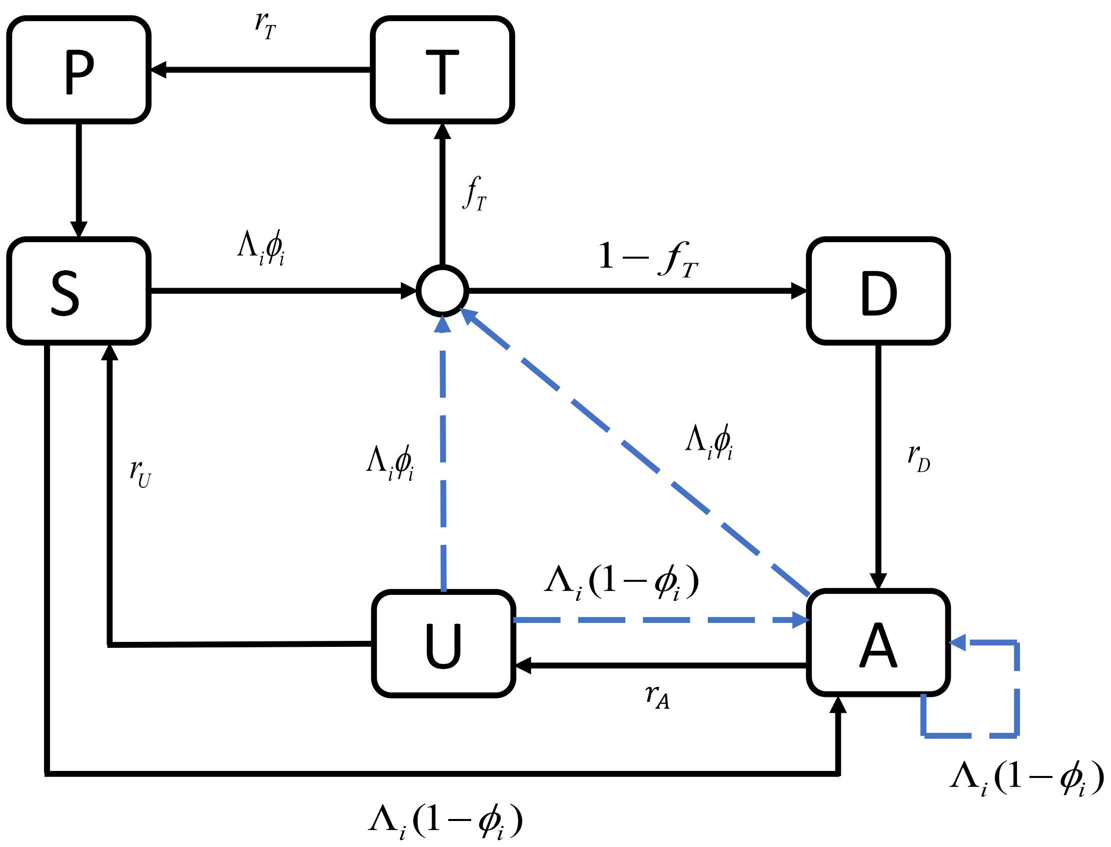

Figure A: Illustration of the progression between human infection states (S = susceptible, D = clinical disease, T = successfully treated disease, P = prophylaxis from prior treatment, A = asymptomatic patent infection, U = asymptomatic subpatent infection). The states are shown in boxes with the transitions marked by arrows and associated hazard rates. The circle represents the treatment node. The dashed blue arrows indication superinfection. Here f_T is the probability of treatment, Λ_i is the force of infection, ϕ_i is the probability of acquiring clinical disease, r are the rates of moving between compartments.

sub-patent infection state (*U*) with rate r_A, before naturally clearing infection and returning to susceptible (*S*) with rate r_U.

Superinfection is included with all age and heterogeneity compartments in states *D*, *A* and *U.* This means that these compartments remain susceptible to re-infection. If this occurs, the proportions of the compartments move into infection states *D*, *T* or *A* in the same process described above. All rates are constant and independent of age.

This model is described by the following coupled ordinary differential equations:

$$\frac{\partial S_i(t)}{\partial t} + \frac{\partial S_i(t)}{\partial a} = -\Lambda_i(t-d_E)S_i(t) + r_P P_i(t) + r_u U_i(t),$$

$$\frac{\partial T_i(t)}{\partial t} + \frac{\partial T_i(t)}{\partial a} = \phi_i f_T \Lambda_i(t - d_E)\big(S_i(t) + A_i(t) + U_i(t)\big) - r_T T_i(t),$$

$$\frac{\partial D_i(t)}{\partial t} + \frac{\partial D_i(t)}{\partial a} = \phi_i (1 - f_T)\Lambda_i(t - d_E)\big(S_i(t) + A_i(t) + U_i(t)\big) - r_D D_i(t), \quad (1)$$

$$\frac{\partial A_i(t)}{\partial t} + \frac{\partial A_i(t)}{\partial a} = (1 - \phi_i)\Lambda_i(t - d_E)\big(S_i(t) + U_i(t)\big) + r_D D_i(t) - \phi_i \Lambda_i(t - d_E) A_i(t) - r_A A_i(t),$$

$$\frac{\partial U_i(t)}{\partial t} + \frac{\partial U_i(t)}{\partial a} = r_A A_i(t) - r_u U_i(t) - \Lambda_i(t - d_E) U_i(t),$$

$$\frac{\partial P_i(t)}{\partial t} + \frac{\partial P_i(t)}{\partial a} = r_T T_i(t) - r_P P_i(t).$$

The parameters for equation (1) are given in Table A. Equations for Λ_i and ϕ_i are evaluated below in equations (4) and (9) respectively.

Table A: The human model parameters for the deterministic model

Parameter	Symbol	Estimate
Latent period	τ_E	12 days
**Rate of leaving human infection stages**		
Asymptomatic patent infection	r_A	0.00512821
Treated clinical disease	r_T	0.2
Untreated clinical disease	r_D	0.2
Asymptomatic patent infection	r_U	0.00906627
Prophylaxis	r_P	0.06666667
**Treatment Parameters**		
Probability of seeking treatment if clinically diseased	f_T	0.4

Heterogeneity in biting rates

Each age compartment is assigned a unique biting rate, ψ_i. This is defined as:

$$\psi_i(a) = 1 - \rho \exp\left(-\frac{a_i}{a_0}\right), \quad (2)$$

for age compartment a_i, where ρ and a_0 are parameters that determine the relationship between age (i.e. body size) and biting rate. The relative biting rate, ζ_j, is drawn from a log-normal distribution with a mean of 1:

$$\log(\zeta_j) \sim N\left(-\frac{\sigma^2}{2}, \sigma^2\right), \quad (1)$$

where j denotes the heterogeneity group of the model.

The entomological inoculation rate (EIR), $\varepsilon_{i,j}$ (or the average number of infecting bites an individual receives), and force of infection, $\Lambda_{i,j}$, experienced by an age compartment i and heterogeneity compartment j, with age a at time t are denoted as:

(4)

$$\varepsilon_{i,j} = \varepsilon_0(t)\zeta_j\psi_i(a),$$

$$\Lambda_{i,j} = b_i(t)\varepsilon_{i,j}(a,t),$$

where $\varepsilon_0(t)$ is the mean EIR experienced by adults at time t, and $b_i(t)$ is the probability that an infectious bite leads to a patent infection, which is determined by the level of pre-erythrocytic immunity and is defined in equation (5). The force of infection is then subject to a lag of τ_E days to account for the latent period of infection.

The parameters for this section are given in Table B. Descriptions for b_i and ε_0 are given in equations (6) and (23).

Table B: Age and heterogeneity parameters for the deterministic malaria model

Parameter	Symbol	Estimate
Age-dependent biting parameter	ρ	0.85
Age-dependent biting parameter	a_0	8 years
Variance of the log heterogeneity in biting rates	σ^2	1.67

Human immunity

The acquisition and loss of naturally-acquired immunity is captured dynamically in the model and is driven by both age and exposure. We consider three transition points at which immunity may act:

1) a reduction in the probability that infection is established following an infectious challenge (pre-erythrocytic immunity, I_B),
2) a reduction in the probability of clinical disease upon infection (clinical immunity, I_C),
3) and a reduction in the detectability of an infection and onward transmission to mosquitoes through blood stage immunity (detection immunity, I_D).

Immunity to infection, $I_{B_{i,j}}(t)$ in a population exposed to an EIR $\varepsilon_{i,j}(t)$ is a function of both age and time and is given by the partial differential equation,

$$\frac{\partial I_{B_{i,j}}}{\partial t} + \frac{\partial I_{B_{i,j}}}{\partial a} = \frac{\varepsilon_{i,j}}{\varepsilon_{i,j} u_B + 1} - \frac{I_{B_{i,j}}}{d_B}, \qquad I_{B_{i,j}}(t) = 0 \tag{2}$$

where u_B limits the rate at which immunity to infection can be boosted at high exposure and d_B is the mean duration of immunity to infection. The probability of infection by age is then given by a Hill function,

$$b_{i,j}(t) = b_0\left(b_1 + \frac{1-b_1}{1+\left(\frac{I_{B_{i,j}}(t)}{I_{B0}}\right)^{\kappa_B}} \right), \tag{3}$$

Where b_0 is the probability of infection with no immunity, $b_0 b_1$ is the minimum probability, I_{B0} and κ_B are scale and shape parameters respectively, and $I_{B_{i,j}}(t)$ is the level of pre-erythrocytic immunity of age compartment i and heterogeneity compartment j at time t.

Immunity to clinical disease, $I_{C_{i,j}}(t)$, comprises of immunity acquired by exposure to infection, $I_{CA_{i,j}}(t)$, and that maternally acquired, $I_{CM_{i,j}}(t)$. $I_{CA_{i,j}}(t)$ in a population exposed to a force of

infection $\Lambda_{i,j}$ is a function of both age, heterogeneity group and time and is described by the partial differential equation:

$$\frac{\partial I_{CA_{i,j}}}{\partial t} + \frac{\partial I_{CA_{i,j}}}{\partial a} = \frac{\Lambda_{i,j}}{\Lambda_{i,j} u_C + 1} - \frac{I_{CA_{i,j}}}{d_{CA}}, \qquad I_{CA_{i,j}}(t) = 0 \tag{4}$$

where u_C limits the rate at which immunity to clinical disease can be boosted at high exposure and d_{CA} is the mean duration of clinical immunity. Maternally-acquired immunity $I_{CM_{i,j}}(t)$ is assumed at birth to be a proportion (P_{CM}) of the level of immunity present in a 20-year old woman, $I_{C_{20,j}}(t)$, living in the same location and which decays at a constant rate $(1/d_M)$,

$$\frac{\partial I_{CM_{i,j}}}{\partial t} + \frac{\partial I_{CM_{i,j}}}{\partial a} = -\frac{I_{CM_{i,j}}}{d_M}, \qquad I_{CM_{i,j}}(t) = P_{CM} I_{C_{20,j}}(t). \tag{5}$$

The total clinical immunity by age and time is given by $I_C = I_{CA} + I_{CM}$. The probability of acquiring clinical disease upon infection by age is then given by a Hill function,

$$\phi_{i,j}(t) = \phi_0 \left(\phi_1 + \frac{1-\phi_1}{1+\left(\frac{I_{CA_{i,j}}(t)+I_{CM_{i,j}}(t)}{I_{C0}}\right)^{\kappa_C}} \right), \tag{6}$$

where ϕ_0 is the probability of disease with no immunity, $\phi_0\phi_1$ is the minimum probability, I_{C0} and κ_C are scale and shape parameters respectively, $I_{CA_{i,j}}(t)$ is the level of acquired immunity to clinical disease and $I_{CM_{i,j}}(t)$ is the level of maternally acquired immunity to clinical disease of age compartment i and heterogeneity class j at time t.

Finally, detection immunity, $I_{D_{i,j}}(t)$, which is the effect of blood stage immunity reducing the detectability of an infection and onward transmission to mosquitoes, is given by the partial differential equation:

$$\frac{\partial I_{D_{i,j}}}{\partial t} + \frac{\partial I_{D_{i,j}}}{\partial a} = \frac{\Lambda_{i,j}}{\Lambda_{i,j} u_D + 1} - \frac{I_{D_{i,j}}}{d_D}, \qquad I_{D_{i,j}}(t) = 0, \tag{7}$$

where u_D limits the rate at which detection immunity can be boosted at high exposure and d_C is the mean duration of detection immunity. The detectability by microscopy of an asymptomatic infection in age compartment i and heterogeneity compartment j at time t is given by:

$$q_{i,j}(t) = d_1 + \frac{1-d_1}{\left(\frac{1+I_{D_{i,j}}(t)}{I_{D0}}\right)^{\kappa_D} f_{D_i}}, \tag{8}$$

where d_1 is the minimum probability of detection, I_{D0} and κ_D are scale and shape parameters respectively, $I_{D_{i,j}}(t)$ is the level of acquired immunity to the detectability of infection of age compartment i and heterogeneity class j at time t, and

$$f_{D_i} = 1 - \frac{1-f_{D0}}{1+\left(\frac{a_i}{a_D}\right)^{\gamma_D}} \tag{9}$$

is an age-dependent (physiological) modifier of the detectability of infection where f_{D0}, a_D and γ_D are parameters.

The immunity parameters are given in Table C.

Table C: Immunity parameters for the deterministic malaria model

Parameter	Symbol	Estimate
**Immunity reducing probability of infection**		
Maximum probability due to no immunity	b_0	0.590076
Maximum relative reduction due to immunity	b_1	0.5
Inverse of decay rate	d_B	10 years
Scale parameter	I_{B0}	43.8787
Shape parameter	κ_B	2.15506
Duration in which immunity is not boosted	u_B	7.19919 days
**Immunity reducing probability of clinical disease**		
Maximum probability due to no immunity	ϕ_0	0.791666
Maximum relative reduction due to immunity	ϕ_1	0.000737
Inverse of decay rate	d_{CA}	30 years
Scale parameter	I_{C0}	18.02366
Shape parameter	κ_C	2.36949
Duration in which immunity is not boosted	u_C	6.06349 days
Inverse of decay rate of maternal immunity	d_M	67.6952 days
New-born immunity relative to mother's	P_{CM}	0.774368
**Immunity reducing probability of detection**		
Minimum probability due to maximum immunity	d_1	0.160527
Inverse of decay rate	d_{ID}	10 years
Scale parameter	I_{D0}	1.577533
Shape parameter	κ_D	0.476614
Duration in which immunity is not boosted	u_D	9.44512 days
Scale parameter relating age to immunity	a_D	21.92 years
Time-scale at which immunity changes with age	f_{D0}	0.007055
Shape parameter relating age to immunity	γ_D	4.8183

Vector model

The larval model is based on the compartmental model previously described in [4]. Female adult mosquitoes (M) lay eggs at rate β_L. Upon hatching from eggs, larvae progress through early and late larvae stages ($E_L$and L_L compartments) before developing into the pupal stage (P_L). The duration spent in each stage is denoted by d_{E_L}, d_{L_L} and d_{P_L} respectively. The larval stages are regulated by density dependent mortalities (μ_{E_L}, μ_{L_L} and μ_{P_L}) with a time-varying carrying-capacity, K_L, which represents the ability of the environment to sustain breeding sites through different periods of the year, and with the density of larvae in relation to the carrying-capacity regulated by a parameter γ_L. The carrying-capacity determines the mosquito density and hence the baseline transmission intensity in the absence of interventions. It is calculated by

$$K_L = M_0 \frac{2 d_{L_L} \mu_0 (1 + d_{P_L} \mu_{P_L}) \gamma_L (\lambda + 1)}{\frac{\lambda}{\mu_{L_L} d_{E_L}} - \frac{1}{\mu_{L_L} d_{L_L}} - 1}, \quad (10)$$

where M_0 is the initial female mosquito density, μ_0 is the baseline mosquito death rate and

$$\lambda = -\frac{1}{2}\left(\gamma_L \frac{\mu_{L_L}}{\mu_{E_L}} - \frac{d_{E_L}}{d_{L_L}} + (\gamma_L - 1)\mu_{L_L} d_{E_L}\right) + \sqrt{\frac{1}{4}\left(\gamma_L \frac{\mu_{L_L}}{\mu_{E_L}} - \frac{d_{E_L}}{d_{L_L}} + (\gamma_L - 1)\mu_{L_L} d_{E_L}\right)^2 + \gamma_L \frac{\nu \mu_{L_L} d_{E_L}}{2\mu_{E_L}\mu_0 d_{L_L}(1+d_{P_L}\mu_{P_L})}}, \quad (11)$$

$$\nu = \frac{\beta_L \mu_M e^{-\frac{\mu_M}{f}}}{\mu_M\left(e^{\frac{\mu_M}{f}} - 1\right)\left(1 - e^{-\frac{\mu_M}{f}}\right)}. \quad (12)$$

Here $\beta_L$is the maximum number of eggs per oviposition per mosquito and

$$\mu_M = -f_R \log(p_1 p_2) \quad (13)$$

where p_1 is the probability of a mosquito surviving one feeding cycle, p_2 is the probability of surviving one resting cycle andf_R is the feeding rate.

The model is described by the equations below:

$$\frac{dE_L}{dt} = \beta_L M - \mu_{E_L}\left(1 + \frac{E_L + L_L}{K_L}\right)E_L - \frac{E_L}{d_{E_L}},$$
$$\frac{dL_L}{dt} = \frac{E_L}{d_{E_L}} - \mu_{L_L}\left(1 + \gamma_L\left(\frac{E_L + L_L}{K_L}\right)\right)L_L - \frac{L_L}{d_{L_L}}, \quad (17)$$
$$\frac{dP_L}{dt} = \frac{L_L}{d_{L_L}} - \mu_{P_L}P_L - \frac{P_L}{d_{P_L}}.$$

We assume 50% of the emergent adult mosquitoes are female and all enter the susceptible state (S_M). These mosquitoes become infected at a rate that depends on the infectiousness of the human population including an appropriate time-lag (τ_G) to account for the time taken for parasites to become infectious gametocytes. The force of infection on mosquitoes, Λ_M, is the sum of the contributions from the different human infection state compartments:

$$\Lambda_M(t) = \frac{\lambda}{\omega}\sum_i \sum_j \zeta_j \psi_i \left(c_D D_{i,j}(t-\tau_G) + c_T T_{i,j}(t-\tau_G) + c_A A_{i,j}(t-\tau_G) + c_U U_{i,j}(t-\tau_G)\right) \quad (14)$$

where ψ_i is the age-dependent biting rate and ζ_j is the relative biting rate for each heterogeneity compartment defined in equations (2) and (3). The parameter λ is the rate at which a person is bitten by mosquitoes, which depends on interventions, and is defined later. The parameter ω represents a normalising constant for the biting rate over all ages:

$$\omega = \int_0^\infty \psi(a) g(a) \mathrm{d}a \quad (15)$$

where $g(a)$ is the human age distribution and is a function of the force of infection for each age group and the proportion of the population in each age compartment at the start of the simulation. The constants c_U, $c_D$and c_T are the infectiousness to mosquitoes from humans in the asymptomatic sub-patent infection, clinical disease and successfully treated compartments and $c_A$is the infectiousness to mosquitoes from humans in the asymptomatic infection compartment, which is calculated as follows:

$$c_{A_{i,j}} = c_U + (c_D - c_U) q_{i,j}{}^{\gamma_1}$$

(16)

where γ_1 is a fitted parameter for the infectiousness of state A and $q_{i,j}$ is the probability of being detected by microscopy, which is given by equation (11).

Once infected, mosquitoes pass through a latent period (E_M) of fixed length τ_M and then they become infectious to humans (I_M). They are assumed to remain infectious until they die. The infection process in the mosquito population is as follows:

$$\frac{dS_M}{dt} = \frac{P_L}{2d_{P_L}} - \Lambda_M S_M - \mu_M S_M,$$

$$\frac{dE_M}{dt} = \Lambda_M S_M - \Lambda_M(t-\tau_M)S_M(t-\tau_M)P_M - \mu_M E_M, \tag{21}$$

$$\frac{dI_M}{dt} = \Lambda_M(t-\tau_M)S_M(t-\tau_M)P_M - \mu_M I_M,$$

where μ_M is the mosquito death rate defined in equation (16) and

$$P_M = e^{-\mu_M \tau_M} \tag{17}$$

is the probability that a mosquito survives the extrinsic incubation period. We define the mean EIR experienced by adults at time t to be:

$$\varepsilon_0(t) = \frac{1}{\omega} I_M(t). \tag{18}$$

The parameters for this part of model are given in Table D.

Table D: Vector model parameters

Parameter	Symbol	Estimate
**Larval model**		
Average number of eggs laid per female mosquito per day	β_L	21.2/day
Early instar larval developmental period	d_{E_L}	6.64 days
Late instar developmental period	d_{L_L}	3.72 days
Pupal developmental period	d_{P_L}	0.643 days
Mortality rate of early-stage larvae (density dependent)	μ_{E_L}	0.0338/day
Mortality rate of late-stage larvae (density dependent)	μ_{L_L}	0.0348/day
Mortality rate of pupae (density independent)	μ_{P_L}	0.249/day
Effect of density dependence on late instars relative to early instars	γ_L	13.25
**Infectiousness to mosquitoes**		
Lag from parasites to infectious gametocytes	τ_G	12.5 days
Untreated disease	c_D	0.068
Treated disease	c_T	0.021896
Sub-patent infection	c_U	0.00062
Parameter for infectiousness of state *A*	γ_1	1.82425
**Mosquito Population Model**		
Baseline daily mortality of adults with no interventions	μ_M	0.132
Mean time between feeds	δ	3 days
Extrinsic incubation period	τ_M	10 days
Initial female mosquito density	M_0	Dependent on EIR
Probability of surviving one feeding attempt	p_1	Intervention dependent
Probability of surviving one resting cycle	p_2	0.737
Mosquito feeding rate	f_R	Intervention dependent
Rate at which a person is bitten by mosquitoes	λ	Intervention dependent

Long-lasting insecticidal nets model

Long-lasting insecticidal nets (LLIN) have four main effects on the transmission cycle:

i) they increase the overall mosquito death rate;

ii) they lengthen the feeding or gonotrophic cycle;

iii) they change the proportion of bites taken on protected and unprotected people;

iv) they change the proportion of bites taken on humans relative to animals (the Human Blood Index).

We model these impacts on the vector population, as in *Griffin et al.* [1]. The probability of a blood-seeking mosquito successfully feeding depends on the behaviour of the mosquito (which may vary between species) and the anti-vectoral defences employed by the human host population.

As shown in the paper, once a mosquito enters a house to feed, one of three things can happen: it can repeat (r_N), feed successfully (s_N) or die (d_N). In the full model with both the barrier effect of the LLIN and insecticide, the repellency of LLIN decreases from a maximum, r_{N0}, to a non-zero level r_{NM}, at a rate $\gamma_N = \log(2)/\eta$ (where η is the half-life of the net) reflecting the protection still provided by a net that no longer has any insecticidal effect (and potentially some holes). The killing effect of LLIN decreases from d_{N0} at the same constant rate. Therefore, at a time t after nets were distributed,

$$\begin{aligned} r_N &= (r_{N0} - r_{NM})\exp(-t\gamma_N) + r_{NM}, \\ d_N &= d_{N0}\exp(-t\gamma_N), \\ s_N &= 1 - r_N - d_N. \end{aligned} \tag{24}$$

These values change with the chemicals used as insecticide and the resistance of the mosquitoes to the chemical [5]. We assume a three-yearly distribution of LLINs with adherence to use decaying over time.

The number of mosquitoes entering a house in search of a blood meal can be estimated from experimental hut trials. The presence of a bed net will cause a mosquito to repeat in one of two ways. First, the mosquito will be less likely to enter a house due to the excito-repellent effect of the insecticide on the nets, and secondly once it enters a house it will be repelled from a protected human due to the physical barrier of the net and the effects of the insecticide.

Not all mosquitoes successfully feed upon entering a house even before the introduction of an intervention. Therefore, the probability of repeating, feeding or dying needs to be relative to that seen in the absence of LLINs. The proportion repeating (r_{N0}), feeding successfully (s_{N0}) and dying (d_{N0}) in the presence of LLIN will therefore be,

$$\begin{aligned} r_{N0} &= \left(1 - \frac{k_1'}{k_0}\right)\left(\frac{j_1'}{j_1' + l_1'}\right), \\ s_{N0} &= \frac{k_1'}{k_0}, \\ d_{N0} &= \left(1 - \frac{k_1'}{k_0}\right)\left(\frac{l_1'}{j_1' + l_1'}\right) \end{aligned} \tag{25}$$

Where $j_1' = \left(1 - \frac{N_1}{N0}\right) + \frac{N_1}{N_0} j_1$, $k_1' = \frac{N_1}{N_0} k_1$ and $l_1' = \frac{N_1}{N_0} l_1$. Here, N_0 and N_1 represent the number of mosquitoes entering a house with or without LLIN. The parameters j_0 and j_1, k_0 and k_1, and l_0 and l_1, denote the percentage of mosquitoes that are not feeding (j) , are successfully feeding (k) or are killed (l) in the absence or presence of bed nets. These associations change with resistance. Principally, the mortality effect r_{N0} is reduced and the half-life of nets γ_N also reduces [5].

The proportion repeating (r_{N0}), feeding successfully (s_{N0}) and dying (d_{N0}) are altered in a setting where local mosquitoes show resistance to pyrethroids. In previous work, we use meta-analyses to estimate the association between the probability of mosquitoes surviving exposure to a discriminatory dose of pyrethroid in the susceptibility bioassay, and the mortality observed in experimental hut trials testing the entomological impact of mosquito nets. We also learn the

associations between the outcome of a mosquito feeding attempt in the experimental hut linking mortality to successful feeding, repellence, and deterrence [6]. We have then estimated the additional benefit from pyrethroid-PBO nets so that we can explore the change in r_{N0}, s_{N0} and d_{N0} with different measures of susceptibility bioassay survival [7]. We have validated this process using a systematic review of randomised control trials as the gold standard method to estimate epidemiological impact of interventions [8]. We use these estimates to explore how resistance impacts on the barrier and community effect provided by the mosquito nets.

Following the same logic, we use the data from Nash et al. [6] to estimate what these parameters are likely to be in the presence of untreated mosquito nets. There were 90 data for untreated nets within the meta-analysis. The median estimate with the 25th and 75th percentiles for the proportion feeding successfully, being killed or being repelled were used to calculate j_1 (0.55, 0.42 – 0.62), k_1 (0.37, 0.26 – 0.55), and l_1 (0.08, 0.03 – 0.14) respectively. Combining these provided estimate for the model parameters r_{N0}, s_{N0} and d_{N0} for untreated nets (Table E)

Table E: LLIN model efficacy parameters used in the deterministic malaria model

**Parameter**	**Symbol**	**Estimate (Treated net)** ***No resistance scenario***	**Estimate (Untreated net)**
Probability of being repelled	r_{N0}	0.563 (0.513 – 0.626)	0.409 (0.538 – 0.198)
Probability of being success feeding	s_{N0}	0.050 (0.036 – 0.060)	0.532 (0.337 – 0.790)
Probability of being killed	d_{N0}	0.387 (0.451 – 0.314)	0.059 (0.125 – 0.012)

We define the probability of a *Plasmodium falciparum* mosquito biting a human host during a single attempt to be y; the probability that a mosquito bites a human host and survives the feeding attempt to be w, and the probability of it being repelled without feeding to be z. These parameters account for the repeating behaviour observed prior to the introduction of insecticides and exclude natural vector mortality. In this model without indoor residual spraying, $y = w$.

During a single feeding attempt (which may be on animals or humans), a mosquito will successfully feed with probability W depending on the intervention usages given by c_k ($k = 1$ is no intervention compartment and if $k = 2$ is LLIN compartment):

$$W = \sum_{k=1}^{2} w_k c_k, \qquad w_k = \begin{cases} 1 & \text{if } k = 1, \\ 1 - \Phi_b + \Phi_b s_N & \text{if } k = 2, \end{cases} \tag{19}$$

and be repelled without feeding with probability Z given by

$$Z = \sum_{k=1}^{2} z_k c_k, \qquad z_k = \begin{cases} 0 & \text{if } k = 1, \\ \Phi_b r_N & \text{if } k = 2, \end{cases} \tag{20}$$

where in both equations Φ_b is the proportion of bites taken on humans in bed and s_N is the probability of successfully feeding upon an encounter with a net and r_N is the probability of repeating. The average probabilities of mosquitos successfully feeding during a single attempt and being repelled without feeding are:

$$\overline{W} = 1 - Q_0 + Q_0 W \tag{21}$$

$$\overline{Z} = Q_0 Z. \tag{22}$$

The proportion of bites taken on humans in bed is defined as

$$,\Phi_B = \frac{\sum_t p_B(t)\lambda_I(t)}{\sum_t\Big(\big(1-p_I(t)\big)\lambda_O(t)+p_I(t)\lambda_I(t)\Big)},$$

where $\lambda_I(t)$ is the rate at which a person who is indoors at hour t is bitten and$\lambda_O(t)$ is the corresponding figure for someone outdoors, and $p_I(t)$ is the proportion of human hosts indoors and $p_B(t)$ in bed at a given time *t.* Due to the lack of data it is assumed that human movement and sleeping patterns are not dependent on age or relative exposure.

The mosquito feeding rate f_R is given by

$$f_R = \frac{1}{\delta_1 + \delta_2}, \tag{23}$$

Where δ_1 and δ_2 are the length of time spent looking for a blood meal and resting between feeds respectively. Parameter δ_2 is assumed to be unaffected by the interventions, whilst δ_1 is increased to

$$\delta_1 = \frac{\delta_{10}}{1-\overline{Z}}, \tag{24}$$

where δ_{10} is the value with no interventions.

The probabilities of surviving the periods of feeding and resting, as mentioned above, are p_1 and p_2. With no interventions,

$$p_{10} = \exp(-\mu_M \delta_{10}), \tag{25}$$

$$p_2 = \exp(-\mu_M \delta_2), \tag{26}$$

where μ_M is the baseline mosquito death rate. With interventions p_2 is unchanged and

$$p_1 = \frac{\overline{W} p_{10}}{1-\overline{Z} p_{10}}. \tag{27}$$

The probability of surviving one feeding cycle is $p_1 p_2$:

$$p_1 p_2 = \exp(-\mu_M / f_R), \tag{28}$$

hence the mosquito death rate, μ_M, is as defined in equation (16). The probability of surviving the extrinsic incubation period, P_M, also changes with intervention usage.

The proportion of successful bites that are on humans depends on the probability of a feeding cycle resulting in a successful bit on a human or an animal. The probability that a feeding cycle ends with a successful bite on a human, q_H, is

$$\begin{gathered} q_H = p_{10}\big(Q_0 W + \overline{Z} q_H\big), \\ q_H = \frac{p_{10} Q_0 W}{1 - \overline{Z} p_{10}}. \end{gathered} \tag{36}$$

The probability that a feeding cycle ends with a successful bite on an animal is,

$$\begin{gathered} q_A = p_{10}\big(1 - Q_0 + \overline{Z} q_A\big), \\ q_A = \frac{p_{10}(1 - Q_0)}{1 - \overline{Z} p_{10}}. \end{gathered} \tag{37}$$

Hence the proportion of successful bites which are on humans is,

$$Q = 1 - \frac{q_A}{q_A + q_H} = 1 - \frac{1 - Q_0}{1 - Q_0 + Q_0 W'} \tag{38}$$

$$= 1 - \frac{1 - Q_0}{\overline{W}}.$$

and the biting rate on humans is,

$$\alpha = Q f_R. \tag{29}$$

This means that the rate at which a person is bitten by a mosquito is:

$$\lambda_k = \frac{\alpha w_k}{W} \tag{30}$$

and the force of infection of humans on mosquitoes is as defined in equation (18).

Considering different intervention compartments adds a third dimension to the ODEs in equation (1). The EIR now varies according to intervention compartment:

$$\varepsilon_{i,j,k} = \frac{I_M}{\omega} \psi_i \zeta_j \lambda_k. \tag{31}$$

The parameters for this model are given in Table F.

Table F: LLIN model parameters used in the deterministic malaria model

**Parameter**	**Symbol**	**Estimate**
Anthropophagy	Q_0	0.92
Proportion of bites taken on humans in bed	Φ_b	0.85
Baseline time spent looking for a blood meal	δ_1	0.69 days
Time spent resting between feeds	δ_2	2.31 days

Supplementary Table 1: Uncertainty parameter ranges used to explore model variation. The untreated and treated net parameters are shown for specified levels of resistance where 0 indicates the absence of pyrethroid resistance (100% of mosquitoes are killed on exposure to a discriminatory dose of pyrethroid insecticide during bioassay testing), 40 and 80 indicate 60% and 20% of mosquitoes are killed on exposure respectively during discriminatory dose bioassay testing. Repellency of mosquitoes from LLINs is also impacted by resistance and is captures by the r_{N0} parameter.

Parameter	Description	Value	Reference
Φ_B	Probability of a mosquito bite in bed in the absence of nets	0.85 (0.53 – 0.98)	Sherrard-Smith et al 2019 [9]
Untreated nets			
d_{N0}	Net efficacy parameter determining the probability of dying	0.059 (0.012 – 0.125)	This paper, derived from Nash [10].
r_{N0}	Net efficacy parameter determining the probability of successfully feeding	0.409 (0.198 – 0.538)	
γ_N	Net efficacy parameter determining half-life (which is included for untreated nets to match the durability of LLINs to reflect an assumption of wear over time).	2.640 (1.616 – 3.000) years	
Treated nets (LLINs)			
d_{N0}	Net efficacy parameter determining the probability of dying	0% resistance: 0.387 (0.202 – 0.451)<br>40% resistance: 0.352 (0.280 – 0.419)<br>80% resistance: 0.270 (0.202 – 0.345)	Sherrard-Smith et al 2022 [7]
r_{N0}	Net efficacy parameter determining the probability of repeating	0% resistance: 0.563 (0.513 – 0.626)<br>40% resistance: 0.568 (0.557 – 0.633)<br>80% resistance: 0.626 (0.575 – 0.662)	
γ_N	Net efficacy parameter determining half-life (to reflect the waning insecticidal impact and wear over time)	0% resistance: 2.640 (1.616 – 3.000) years<br>40% resistance: 2.226 (1.662 – 2.498) years<br>80% resistance: 1.616 (1.189 – 1.823) year	

Supplementary Table 2: DHS Surveys used in analysis

Country	Code	Years
Angola	AO	2015
Benin	BJ	2012, 2017
Burkina Faso	BF	2010, 2014, 2017
Burundi	BU	2012
DRC	CD	2013
Cote d'Ivoire	CI	2012
Ghana	GH	2014, 2016, 2019
Guinea	GN	2012
Kenya	KE	2015
Liberia	LB	2011, 2016
Madagascar	MD	2011, 2013, 2016
Malawi	MW	2012, 2014, 2017
Mali	ML	2012, 2015, 2018
Mozambique	MZ	2011, 2015, 2018
Nigeria	NG	2010, 2015, 2018
Rwanda	RW	2010, 2015
Senegal	SN	2010, 2012, 2014, 2015, 2016
Sierra Leone	SL	2016
Tanzania	TZ	2012, 2015, 2017
Togo	TG	2013, 2017
Uganda	UG	2014, 2016, 2018

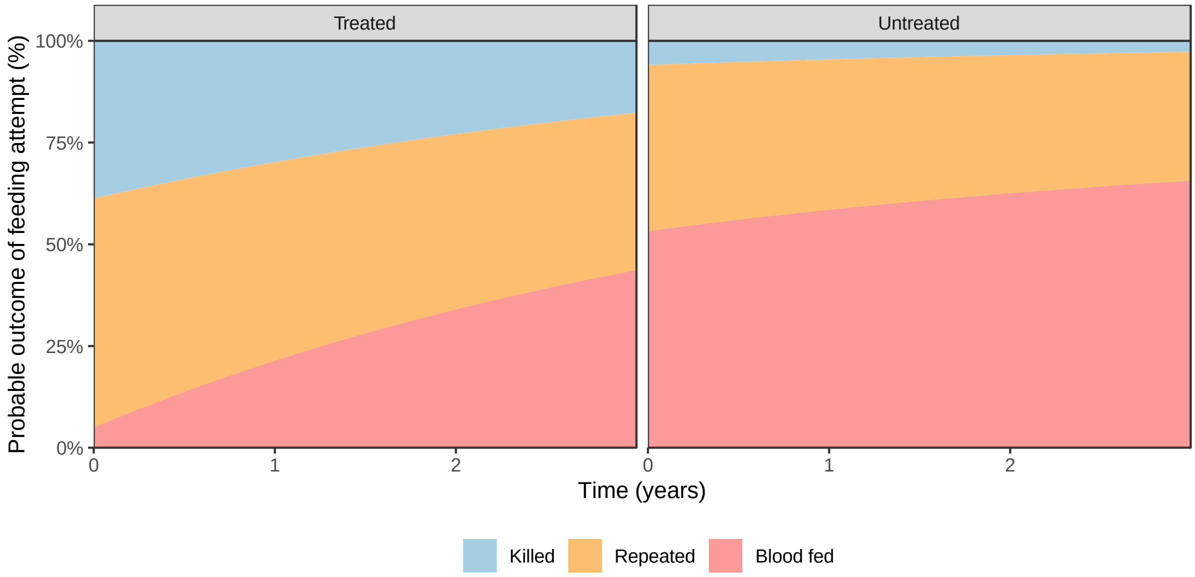

Supplementary Fig. 1: Changes in probable outcomes of feeding attempts for Treated and Untreated nets. The red area shows the percentage of mosquitoes that will die, green that will repeat and blue that will successfully feed. Models were fit to data summarised by Nash et al. using the parameter estimates given in Supplementary Table 1.

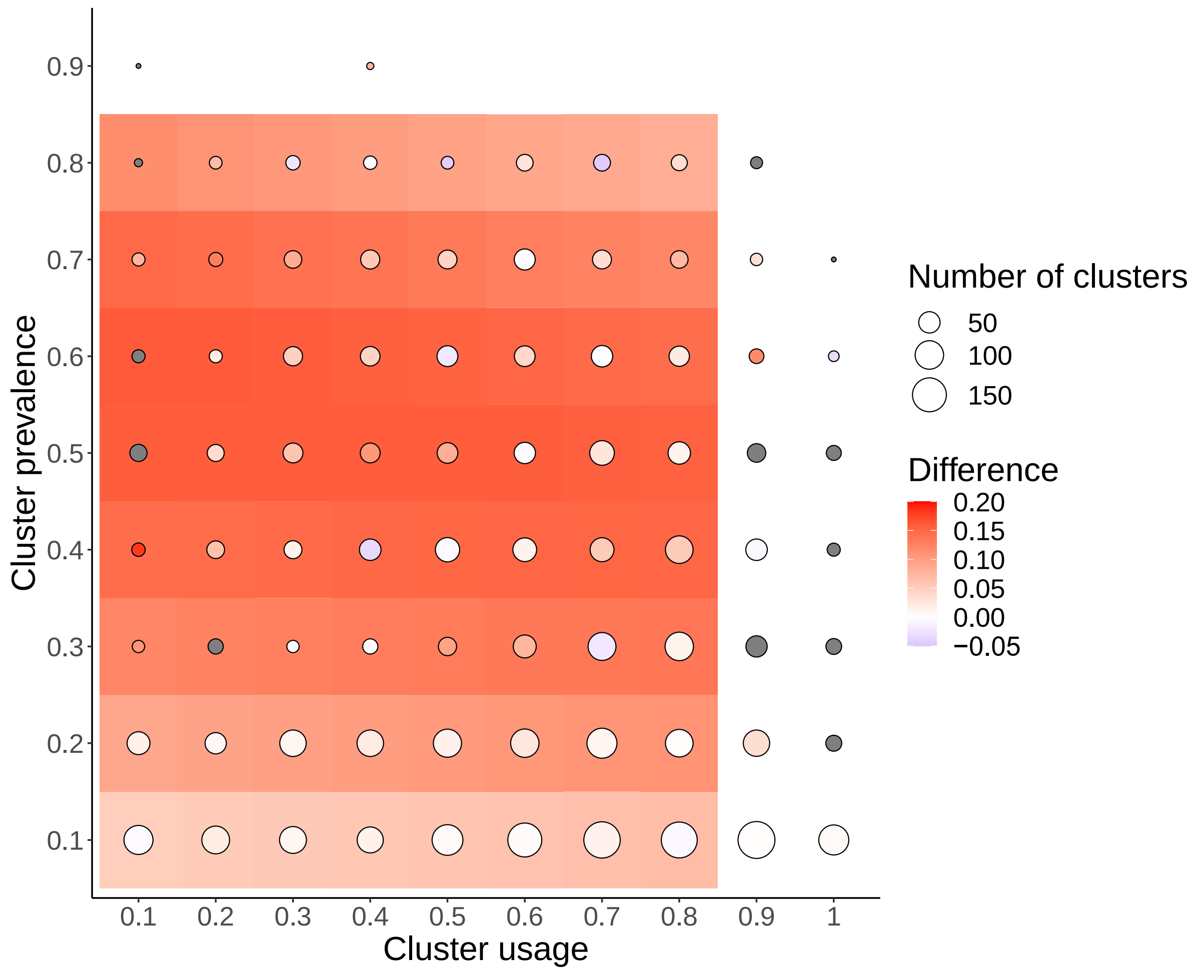

Supplementary Fig. 2: **The absolute difference in prevalence between long lasting insecticidal net (LLIN) users and non-users aged 6-59-months from the DHS survey** (points, size represents number of data points) and model estimates (tiles). This figure is similar to Figure 1 but includes more data that differs, as explained, from the model. Squares with no point represent cluster usage prevalence combinations that were missing from the data and grey circles show where a negative difference was obtained. White squares show high usage and prevalences where the model was not run as it is difficult to calibrate.

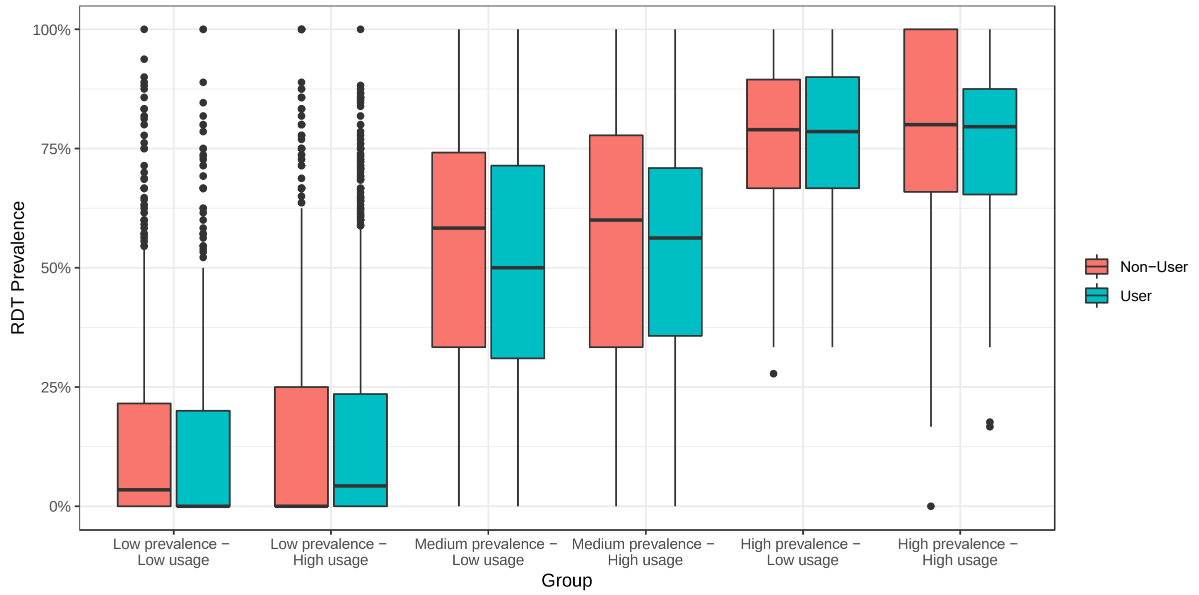

Supplementary Fig. 3: Rapid diagnostic test (RDT) prevalence for DHS data grouped according to cluster usage and prevalence. Clusters were grouped into low (0-33%), medium (33-66%) and high (66-100%) malaria prevalence and low (20-50%) and high (50-80%) net usage. 2972 samples were used each for users and non-users. The centre of the box and whisker plots shows the median, outside lines showing the first and third quartiles, and the whiskers indicate 1.5 times the interquartile range. Dots indicate outliers.

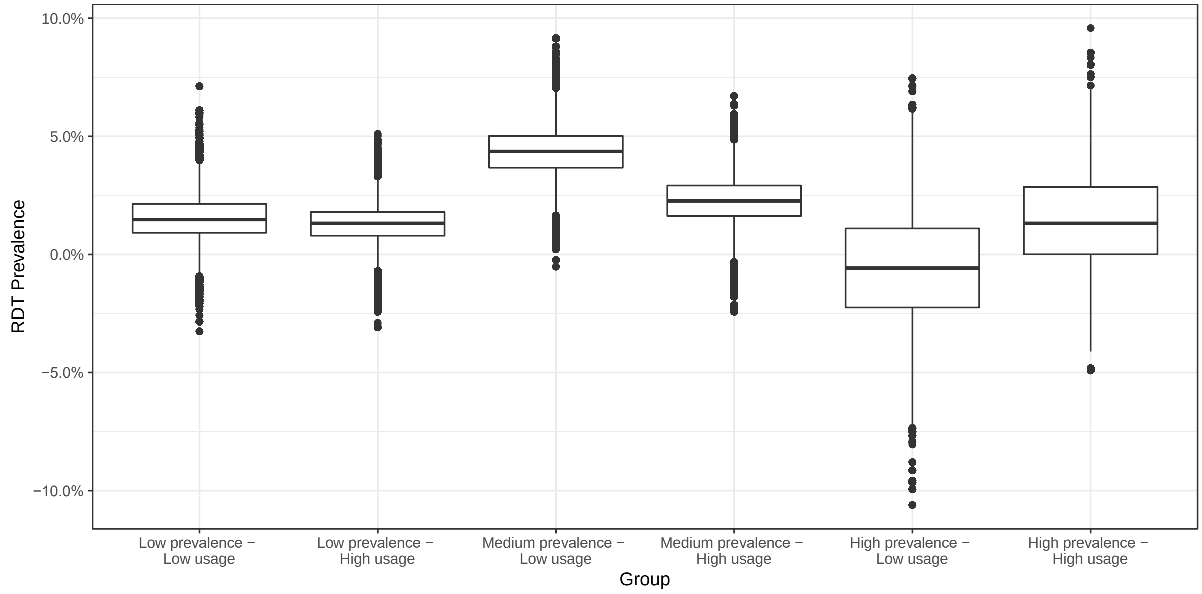

Supplementary Fig. 4: Statistical model estimates for rapid diagnostic test (RDT) prevalence fitted to data in Supplementary Fig. 3. Clusters were grouped into low (0-33%), medium (33-66%) and high (66-100%) malaria prevalence and low (20-50%) and high (50-80%) net usage. Uncertainty is calculated by taking 1000 samples using the bootMer function from the lmer4 package. 2,972,000 samples were used to generate each box. The centre of the box and whisker plots shows the median, outside lines showing the first and third quartiles, and the whiskers indicate 1.5 times the interquartile range. Dots indicate outliers.

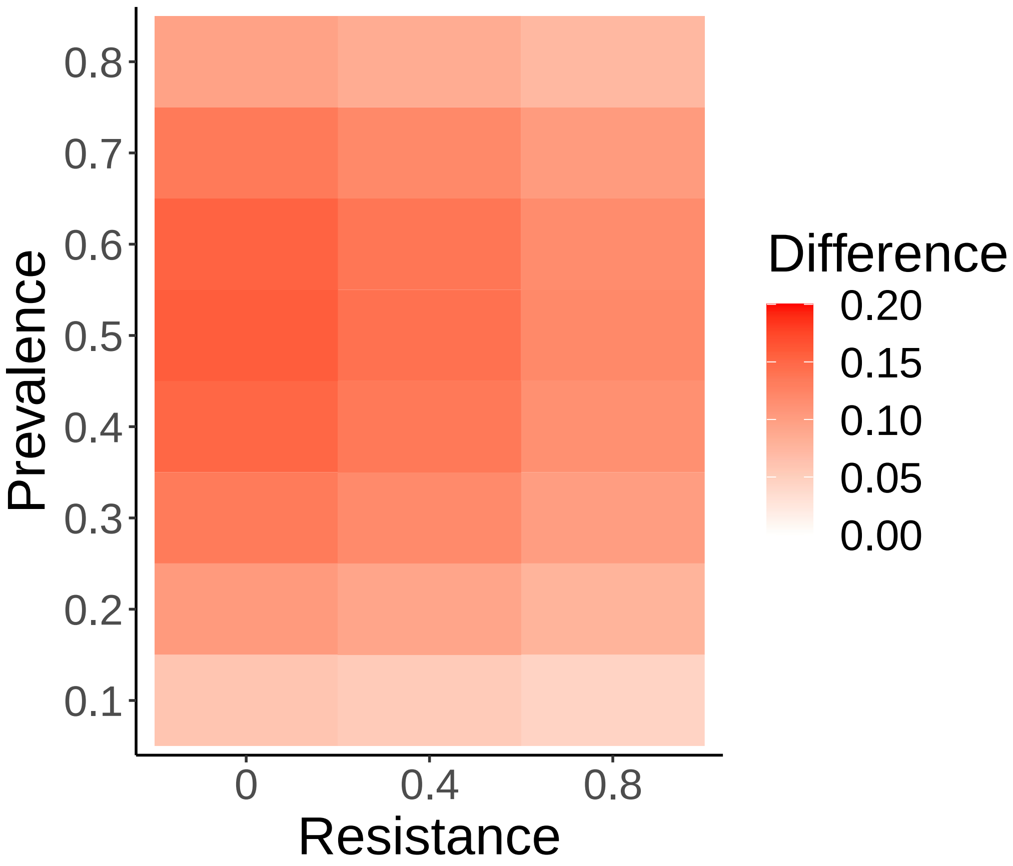

Supplementary Fig. 5. Transmission model estimates of the absolute difference in prevalence between users and non-users of long lasting insecticidal nets (LLINs) for varying endemicities of disease (slide prevalence) and levels of pyrethroid resistance in the local mosquito population. An overall LLIN usage of 50% is assumed throughout. The level of resistance assumed by the model is measured as the percentage of mosquitoes surviving a discriminating dose bioassay.

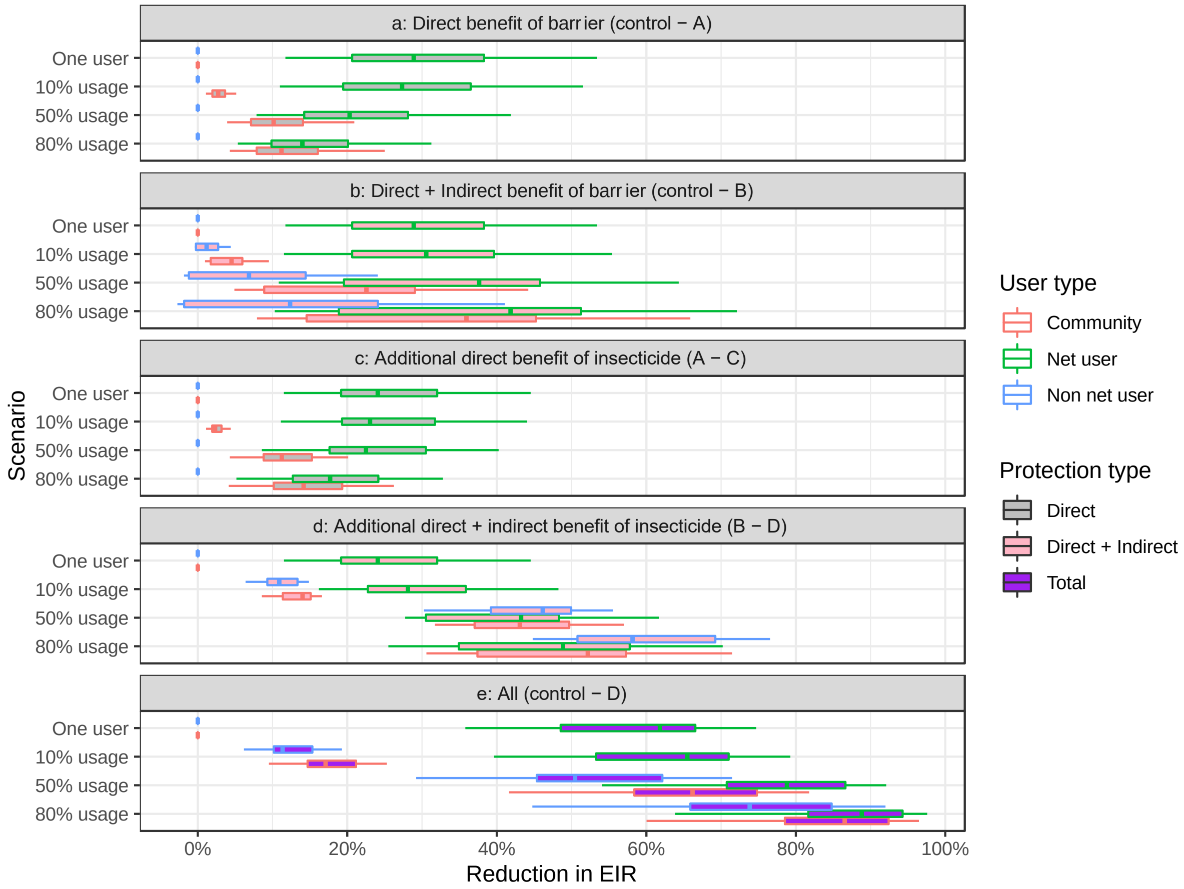

Supplementary Fig. 6. Relative reduction in entomological inoculation rate (EIR) from direct and direct + mass community (indirect) protection offered by mosquito nets for a pre-intervention EIR of 100. The reduction in EIR is calculated from a control, where nobody in the population is given a net, for the 5 scenarios detailed in the methods section. Scenarios (A) – (D) (Figure 2) are repeated for an individual using a net and 10%, 50% and 80% of the population using nets. The 5 subplots (a-e) show results for a different type of protection. Results are the same as Figure 3 of the main text, but here direct and indirect benefits are added together to illustrate the overall protection provided by the different phenomenon. Panel b highlights how the benefit of untreated nets changes with long lasting insecticidal net (LLIN) usage, whilst panel e is identical to the Figure 3e. The reduction for users is shown in green, non-users in blue and the whole community in red. Direct reductions in EIR are filled in grey, direct + indirect reductions in pink and total reductions in purple. The total protection offered by an untreated net is shown here as the direct + indirect benefit of barrier. Box-plots show the range of uncertainty generated by the sensitivity analyses with the centre line indicating median, box limits as the upper and lower quartiles and whiskers 1.5x interquartile range. 15 samples were used to generate each box.

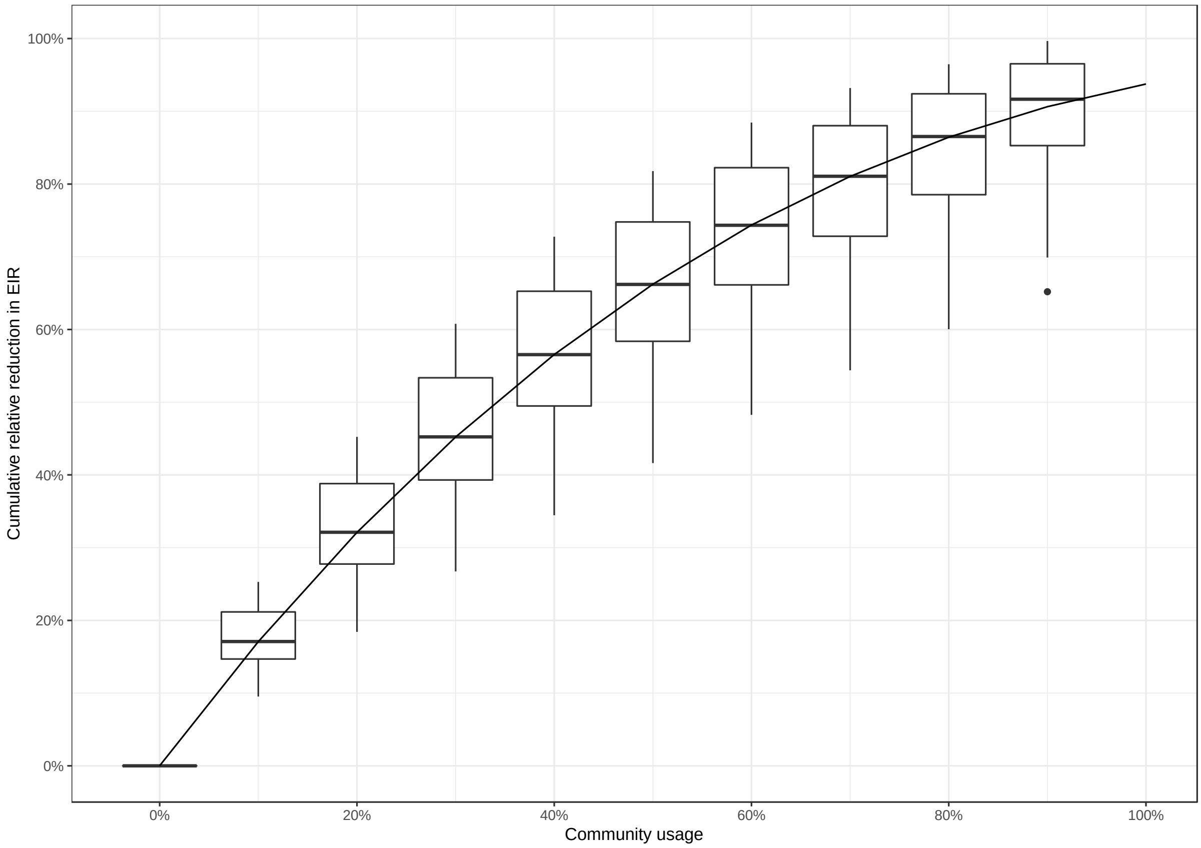

Supplementary Fig. 7: Cumulative reduction in community entomological inoculation rate (EIR) at different usage levels. The centre line of the box plot indicates the median, box limits as the upper and lower quartiles and whiskers 1.5x interquartile range. 15 samples were used to generate each box.

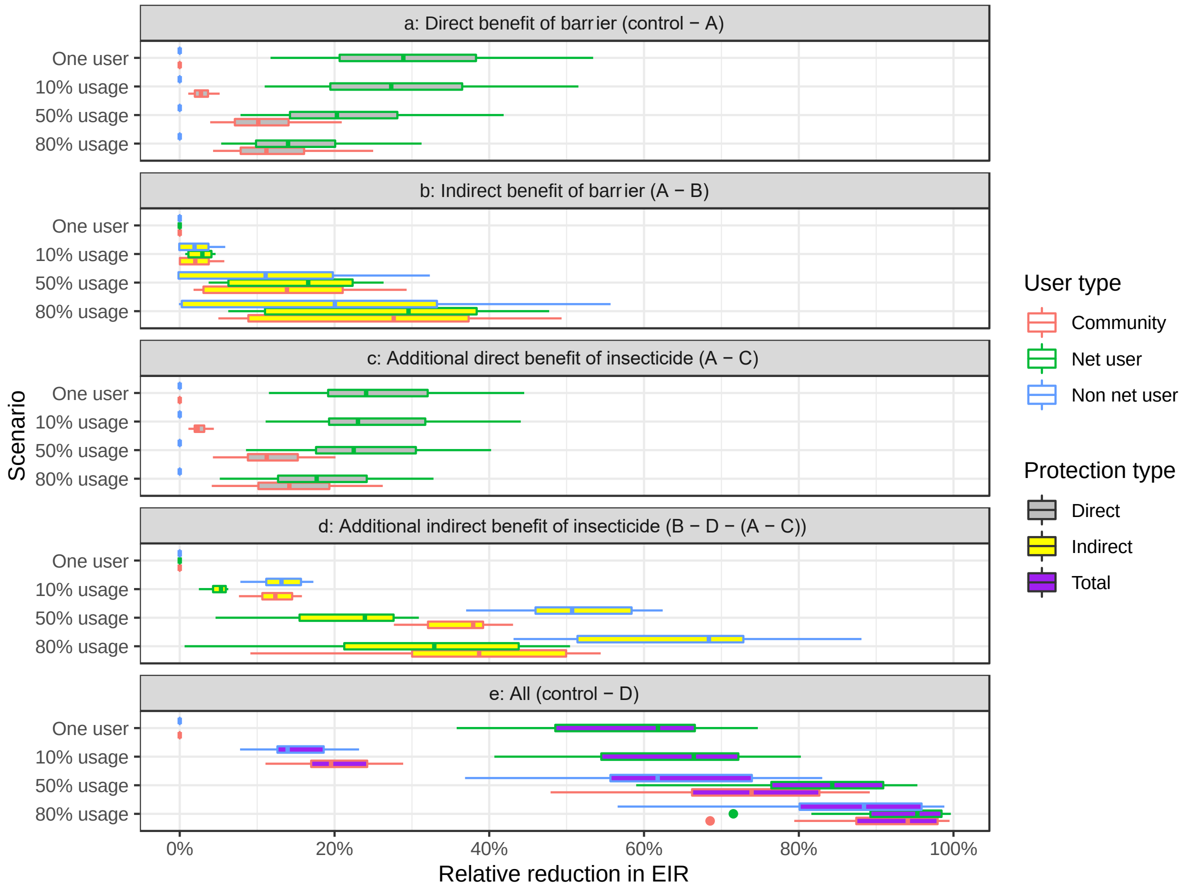

Supplementary Fig. 8: Relative reduction in entomological inoculation rate (EIR) from direct and mass community (indirect) protection offered by mosquito nets for a pre-intervention EIR of 10. The reduction in EIR is calculated relative to a control scenario, where nobody in the population is given a net, for the 5 scenarios detailed in the methods section. Scenarios (A) – (D) (Figure 2) are repeated for an individual using a net and 10%, 50% and 80% of the population using nets. The 5 subplots (a-e) show results for a different type of protection. The reduction for users is shown in green, non-users in blue and the whole community in red. Direct reductions in EIR are filled in grey, indirect reductions in yellow and total reductions in purple. Box-plots show the range of uncertainty generated by the sensitivity analyses with the centre line indicating median, box limits as the upper and lower quartiles and whiskers 1.5x interquartile range. 15 samples were used to generate each box.

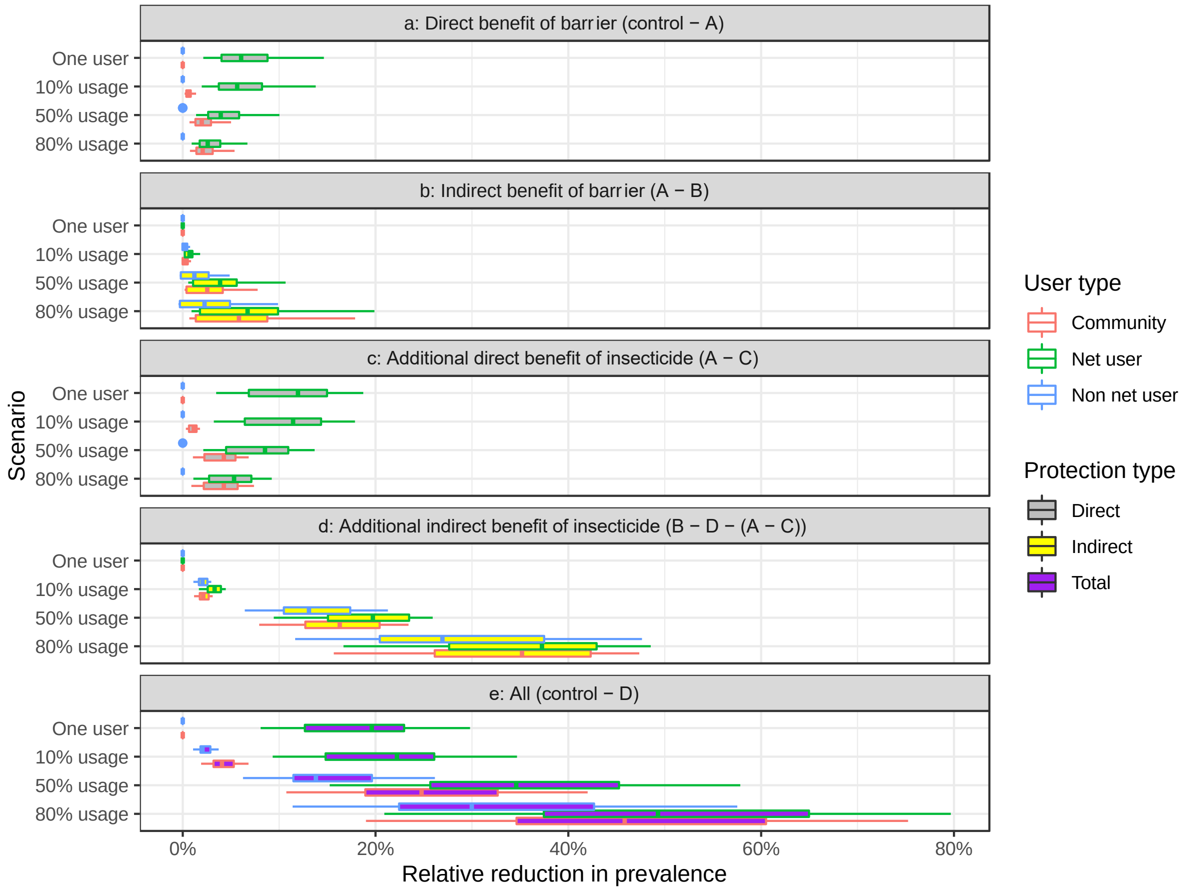

Supplementary Fig. 9: Relative reduction in prevalence from direct and mass community (indirect) protection offered by mosquito nets for a pre-intervention entomological inoculation rate (EIR) of 100. The reduction in prevalence is calculated from a control, where nobody in the population is given a net, for the 5 scenarios detailed in the methods section. Scenarios (A) – (D) (Figure 2) are repeated for an individual using a net and for 10%, 50% and 80% of the population using nets. The 5 subplots (a-e) show results for a different type of protection. The reduction for users is shown in green, non-users in blue and the whole community in red. Direct reductions in EIR are filled in grey, indirect reductions in yellow and total reductions in purple. Box-plots show the range of uncertainty generated by the sensitivity analyses with the centre line indicating median, box limits as the upper and lower quartiles and whiskers 1.5x interquartile range. 15 samples were used to generate each box.

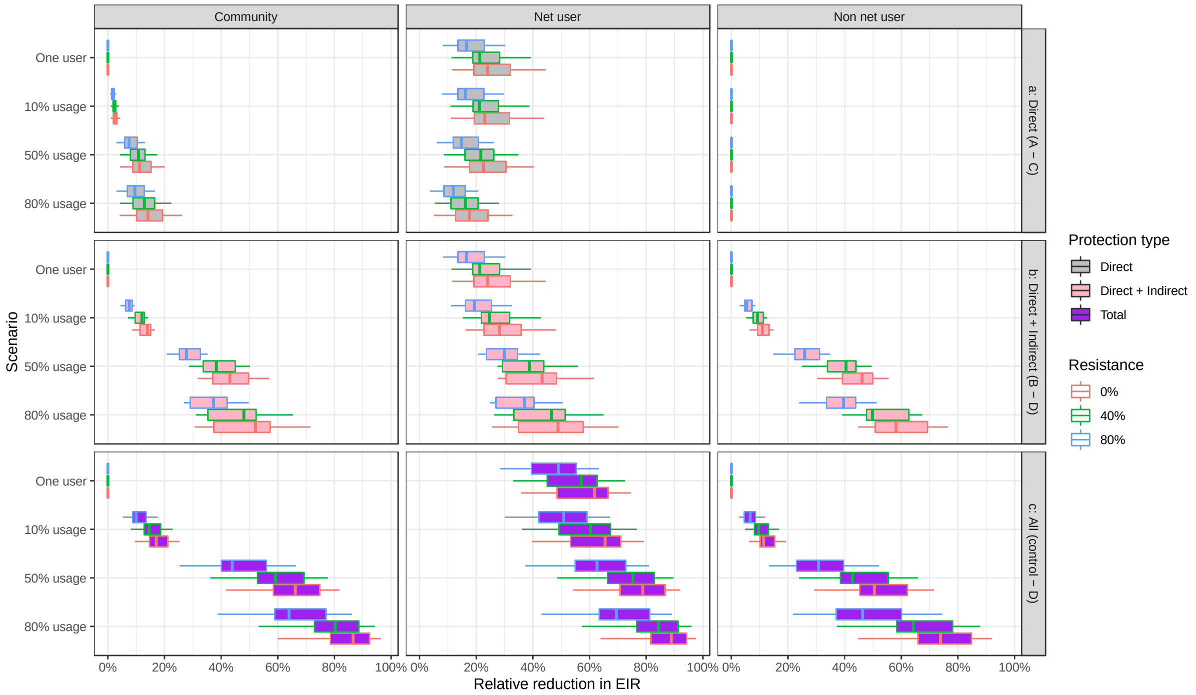

Supplementary Fig. 10: Relative reduction in Entomological inoculation rate (EIR) from direct and direct + indirect protection offered by long lasting insecticidal nets (LLINs) at differing levels of pyrethroid resistance for a pre-intervention EIR of 100. Left figure shows the effect for the community, middle figure shows the effect for users and right figure shows the effect for non-users. Rows (a-c) indicate type of protection offered. The reduction in EIR is calculated from a control, where nobody in the population is given a net, for the scenarios detailed in the methods section where insecticide is included. Usages of 1 person in the population, 10%, 50% and 80% are considered for 0% resistance (as in Figure 3) and 40% and 80% resistance. The barrier only effect remains constant at differing levels of resistance so are not included. The letters (A)-(D) correspond to the scenarios in Figure 2. Box-plots show the range of uncertainty generated by the sensitivity analyses with the centre line indicating median, box limits as the upper and lower quartiles and whiskers 1.5x interquartile range. 15 samples were used to generate each box.

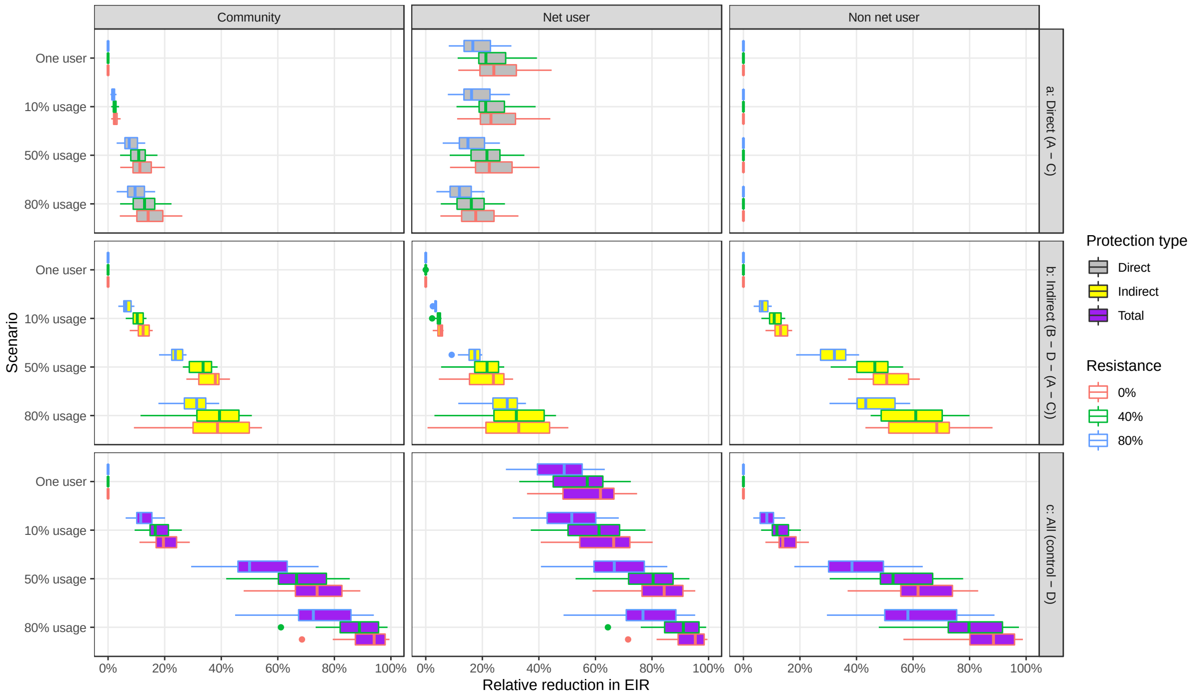

Supplementary Fig. 11: Relative reduction in entomological inoculation rate (EIR) from mass community protection offered by long lasting insecticidal nets (LLINs) at differing levels of pyrethroid resistance for a pre-intervention EIR of 10. Left figure shows the effect for the community, middle figure shows the effect for users and right figure shows the effect for non-users. Rows (a-c) indicate type of protection offered. The reduction in EIR is calculated from a control, where nobody in the population is given a net, for the scenarios detailed in the methods section where insecticide is included. Usages of 1 person in the population, 10%, 50% and 80% are considered for 0% resistance (as in Figure 3) and 40% and 80% resistance. The barrier only effect remains constant at differing levels of resistance so are not included. The letters (A)-(D) correspond to the scenarios in Figure 2. Box-plots show the range of uncertainty generated by the sensitivity analyses with the centre line indicating median, box limits as the upper and lower quartiles and whiskers 1.5x interquartile range. 15 samples were used to generate each box.

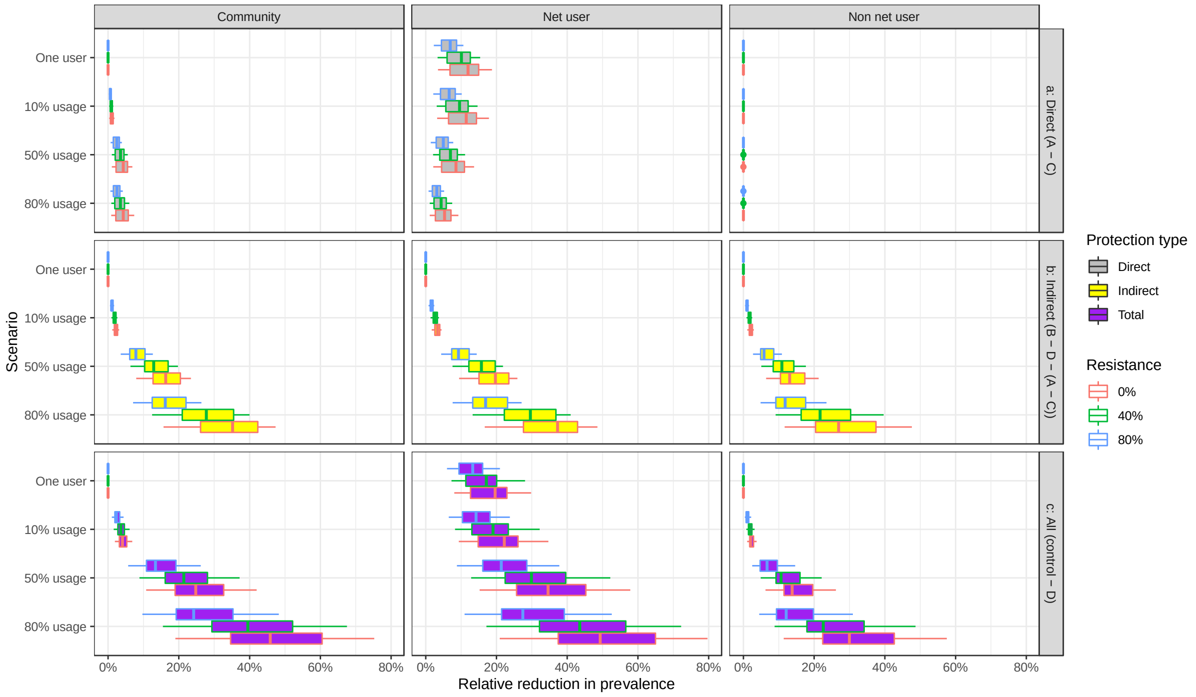

Supplementary Fig. 12: Relative reduction in prevalence from direct and mass community (indirect) protection offered by long lasting insecticidal nets (LLINs) at differing levels of pyrethroid resistance for a pre-intervention Entomological inoculation rate (EIR) of 100. Left figure shows the effect for the community, middle figure shows the effect for users and right figure shows the effect for non-users. Rows (a-c) indicate type of protection offered. The reduction in prevalence is calculated from a control, where nobody in the population is given a net, for the scenarios detailed in the methods section where insecticide is included. Usages of 1 person in the population, 10%, 50% and 80% are considered for 0% resistance (as in Supplementary Fig. 5) and 40% and 80% resistance. The barrier only effect remains constant at differing levels of resistance so are not included. The letters (A)-(D) correspond to the scenarios in Figure 2. Box-plots show the range of uncertainty generated by the sensitivity analyses with the centre line indicating median, box limits as the upper and lower quartiles and whiskers 1.5x interquartile range. 15 samples were used to generate each box.

References

[1] Griffin JT, Hollingsworth TD, Okell LC, et al. Reducing Plasmodium falciparum malaria transmission in Africa: a model-based evaluation of intervention strategies. *PLoS Med* 2010; 7: e1000324.

[2] Winskill P, Slater HC, Griffin JT, et al. The US President's Malaria Initiative, Plasmodium falciparum transmission and mortality: A modelling study. *PLOS Med* 2017; 14: e1002448.

[3] Okell LC, Cairns M, Griffin JT, et al. Contrasting benefits of different artemisinin combination therapies as first-line malaria treatments using model-based cost-effectiveness analysis. *Nat Commun* 2014; 5: 5606.

[4] White MT, Griffin JT, Churcher TS, et al. Modelling the impact of vector control interventions on Anopheles gambiae population dynamics. *Parasit Vectors* 2011; 4: 153.

[5] Churcher TS, Lissenden N, Griffin JT, et al. The impact of pyrethroid resistance on the efficacy

and effectiveness of bednets for malaria control in Africa. *Elife*; 5. DOI: 10.7554/eLife.16090.

[6] Nash RK, Lambert B, N'Guessan R, et al. Systematic review of the entomological impact of insecticide-treated nets evaluated using experimental hut trials in Africa. *Curr Res Parasitol Vector-Borne Dis* 2021; 1: 100047.

[7] Sherrard-Smith E, Winskill P, Hamlet A, et al. Optimising the deployment of vector control tools against malaria: a data-informed modelling study. *Lancet Planet Heal*. DOI: 10.1016/S2542-5196(21)00296-5.

[8] Sherrard-Smith E, Ngufor C, Sanou A, et al. Inferring the epidemiological benefit of indoor vector control interventions against malaria from mosquito data. *Nat Commun* 2022; 13: 3862.

[9] Sherrard-Smith E, Skarp JE, Beale AD, et al. Mosquito feeding behavior and how it influences residual malaria transmission across Africa. *Proc Natl Acad Sci U S A* 2019; 201820646.

[10] Nash RK, Lambert B, N'Guessan R, et al. Systematic review of the entomological impact of insecticide-treated nets evaluated using experimental hut trials in Africa. *Curr Res Parasitol Vector-Borne Dis* 2021; 1: 100047.
