# Supplementary material for: Quantifying the direct and indirect protection provided by insecticide treated bed nets against malaria
Source: Nat Commun. 2023 Feb 8;14:676. doi: 10.1038/s41467-023-36356-9 (PMC9905482; doi:10.1038/s41467-023-36356-9)
Supplement: Supplementary file 3 — Reporting Summary [file 41467_2023_36356_MOESM3_ESM.pdf]

nature portfolio

Corresponding author(s): HJT Unwin

Last updated by author(s): Jan 24, 2022

Reporting Summary

Nature Portfolio wishes to improve the reproducibility of the work that we publish. This form provides structure for consistency and transparency in reporting. For further information on Nature Portfolio policies, see our Editorial Policies and the Editorial Policy Checklist.

Statistics

For all statistical analyses, confirm that the following items are present in the figure legend, table legend, main text, or Methods section.

n/a	Confirmed	
☐	☒	The exact sample size (*n*) for each experimental group/condition, given as a discrete number and unit of measurement
☒	☐	A statement on whether measurements were taken from distinct samples or whether the same sample was measured repeatedly
☐	☒	The statistical test(s) used AND whether they are one- or two-sided<br>*Only common tests should be described solely by name; describe more complex techniques in the Methods section.*
☐	☒	A description of all covariates tested
☒	☐	A description of any assumptions or corrections, such as tests of normality and adjustment for multiple comparisons
☐	☒	A full description of the statistical parameters including central tendency (e.g. means) or other basic estimates (e.g. regression coefficient) AND variation (e.g. standard deviation) or associated estimates of uncertainty (e.g. confidence intervals)
☐	☒	For null hypothesis testing, the test statistic (e.g. *F*, *t*, *r*) with confidence intervals, effect sizes, degrees of freedom and *P* value noted<br>*Give P values as exact values whenever suitable.*
☒	☐	For Bayesian analysis, information on the choice of priors and Markov chain Monte Carlo settings
☒	☐	For hierarchical and complex designs, identification of the appropriate level for tests and full reporting of outcomes
☒	☐	Estimates of effect sizes (e.g. Cohen's *d*, Pearson's *r*), indicating how they were calculated

Our web collection on statistics for biologists contains articles on many of the points above.

Software and code

Policy information about availability of computer code

Data collection	No software was used for data collection.
Data analysis	All analysis was done using R version 3. The deterministic malaria model code can be found at https://github.com/mrc-ide/deterministic-malaria-model with the analysis scripts located at https://github.com/ettieunwin/direct_indirect_bednet_protection. The R packages rdhs version 0.7.4 was used to extract the DHS data from the website and lme4 verson 1.1-27.1 for our regression analysis.

For manuscripts utilizing custom algorithms or software that are central to the research but not yet described in published literature, software must be made available to editors and reviewers. We strongly encourage code deposition in a community repository (e.g. GitHub). See the Nature Portfolio guidelines for submitting code & software for further information.

Data

Policy information about availability of data

All manuscripts must include a data availability statement. This statement should provide the following information, where applicable:
- Accession codes, unique identifiers, or web links for publicly available datasets
- A description of any restrictions on data availability
- For clinical datasets or third party data, please ensure that the statement adheres to our policy

The Demographic Health Survey data is publicly available at https://dhsprogram.com with scripts included on GitHub to access the exact surveys.

Human research participants

Policy information about studies involving human research participants and Sex and Gender in Research.

Reporting on sex and gender	Our analysis was not disaggregated by sex and gender because our model does not consider this.
Population characteristics	We considered children aged 6-59 months in our DHS analysis and all aged population for our model estimates.
Recruitment	We did not recruit anyone for this study. We only analyzed secondary data.
Ethics oversight	No ethics was needed as only used publicly available secondary data

Note that full information on the approval of the study protocol must also be provided in the manuscript.

Field-specific reporting

Please select the one below that is the best fit for your research. If you are not sure, read the appropriate sections before making your selection.

☒ Life sciences ☐ Behavioural & social sciences ☐ Ecological, evolutionary & environmental sciences

For a reference copy of the document with all sections, see nature.com/documents/nr-reporting-summary-flat.pdf

Life sciences study design

All studies must disclose on these points even when the disclosure is negative.

Sample size	We used all publicly available demographic health survey (DHS) data available for African countries since 2010 with geolocated cluster data, Plasmodium falciparum malaria prevalence and LLIN usage.
Data exclusions	We exclude usage and RDT outcomes from clusters with fewer than 20 children, which results in the inclusion of 111,250 individuals of 6-59-months of age in 4,138 clusters.
Replication	We use a deterministic model and include uncertainty intervals from investigating different parameter combinations.
Randomization	Not applicable as no groups were used.
Blinding	Not applicable as no groups were used.

Reporting for specific materials, systems and methods

We require information from authors about some types of materials, experimental systems and methods used in many studies. Here, indicate whether each material, system or method listed is relevant to your study. If you are not sure if a list item applies to your research, read the appropriate section before selecting a response.

Materials & experimental systems

n/a	Involved in the study	
☒	☐	Antibodies
☒	☐	Eukaryotic cell lines
☒	☐	Palaeontology and archaeology
☒	☐	Animals and other organisms
☒	☐	Clinical data
☒	☐	Dual use research of concern

Methods

n/a	Involved in the study	
☒	☐	ChIP-seq
☒	☐	Flow cytometry
☒	☐	MRI-based neuroimaging
